# Determinants of Behavioral Changes Since COVID-19 among Middle School Students

**DOI:** 10.3390/healthcare9010075

**Published:** 2021-01-14

**Authors:** Jaewon Lee, Jennifer Allen, Hyejung Lim, Gyuhyun Choi

**Affiliations:** 1 Department of Social Welfare, Inha University, Incheon 22212, Korea; j343@inha.ac.kr; 2 School of Social Work, Michigan State University, East Lansing, MI 48823, USA; allenj66@msu.edu; 3 School of Education, Korea University, Seoul 02841, Korea; 4 Integrative Arts Therapy, Dongduk Women’s University, Seoul 02748, Korea; toyou4048@uos.ac.kr

**Keywords:** COVID-19, protective behavior changes, individual, family, environmental factor

## Abstract

Middle school students are of particular interest when examining the impact of the COVID-19 pandemic because they are in a formative period for socioemotional development, and because they are not as mature as adults, making them more vulnerable to the effects of the current pandemic. This study seeks to examine determinants of protective behavior changes since COVID-19 among middle school students. Participants were recruited through an official online flatform used by public schools. The final sample included 328 middle school students in South Korea. A multiple linear regression was conducted to explore what factors influence protective behavior changes since COVID-19. Gender and health status were associated with protective behavior changes since COVID-19. Family satisfaction was positively associated with protective behavior changes. Levels of sanitation since COVID-19 and perceptions regarding the risk of COVID-19 were significantly related to protective behavior changes. This study suggests to consider three factors–individual, family, and environmental—in order to prevent middle school students from contracting and spreading the virus.

## 1. Introduction

As of mid-December 2020, the World Health Organization has reported nearly 74 million confirmed cases of COVID-19 and 1.6 million associated deaths worldwide [1]. In South Korea, there have been 48,570 confirmed cases and 659 associated deaths since COVID-19 emerged in Wuhan, China, in December 2019, and was identified as a new coronavirus in January 2020 [1,2]. By mid-February 2020, the number of cases in South Korea began to spread quickly, with the outbreak centered in the city of Daegu, southeast of Seoul [3]. The South Korean government implemented protective measures against the spread of COVID-19, including a national infectious disease plan stemming from the 2015 MERS outbreak; nationwide contact tracing efforts; and a ban on the export of face masks [4,5]. Such policies that encourage protective behaviors such as social distancing have been found to be effective in reducing the spread of COVID-19 [6]. Many of these social distancing measures, such as school closures and the subsequent transition to online learning, greatly affect adolescents, and researchers have found that the continued spread of COVID-19 has many deleterious effects on adolescents’ mental, physical, and socioemotional health [7,8,9,10,11,12,13]. Therefore, it is of interest to examine the extent to which adolescents engage in protective behaviors against the spread of COVID-19, such as staying home and being more diligent about hygiene, and what determinants influence such behaviors, so that the spread of COVID-19 may be reduced [14].

### 1.1. Literature Review

#### 1.1.1. Impacts of the COVID-19 Pandemic on Adolescents

Emerging research has shown that since the COVID-19 pandemic emerged, adolescents are at increased risk for various mental and physical health issues [7,10,11,13,15,16]. In a longitudinal study of adolescents in Shanghai, China conducted from January to March 2020, adolescents’ physical activity decreased significantly, from an average of 540 min per week to 105 min per week [13]. In a 22-week longitudinal study of Australian adolescents, there was also a significant decrease in physical activity after the government of New South Wales implemented social distancing policies [17]. Similar findings were also reported in a sample of adolescents in southern Croatia, with physical activity levels particularly decreasing among boys [16].

Adolescents are also at increased risk for negative mental health outcomes since the beginning of the COVID-19 pandemic [10,11,15]. A review of the preliminary literature indicated an increased risk of posttraumatic stress disorder, depressive and anxiety symptoms among adolescents since the pandemic emerged [11]. Additionally, in a longitudinal study with Norwegian adolescents, the prevalence of mental distress increased significantly from February 2019 to June 2020 [15]. Moreover, in a survey of Canadian adolescents, stress related to the pandemic was associated with increased feelings of loneliness and depression [10].

#### 1.1.2. Determinants of Adolescents’ Protective Behavior Changes Since COVID-19

Utilizing Bronfenbrenner’s ecological perspective on human development, determinants at multiple levels of the ecological system (e.g., individual and family) impact adolescents’ protective behavior changes since COVID-19 [18]. Such protective changes may include increased handwashing; wearing a face mask; keeping distance from others; and working or attending school from home [14]. One determinant of protective behavior changes among adolescents that has emerged in the literature at the individual level is gender [16,19,20,21,22,23]. Compared to their female counterparts, male adolescents in Norway, Poland and Jordan, and young adult men in Switzerland were less likely to report protective handwashing behaviors [19,20,21,23]. Further, female adolescents in Poland and young adult women in Switzerland reported higher compliance with social distancing behaviors than their male counterparts, while no statistically significant difference by gender was found in a sample of adolescents in the United States [20,21,22]. Across studies, females were also found to be more likely than males to use hand sanitizer, avoid touching their face, and wear a face mask [19,20,21].

Another determinant of protective behaviors is one’s perception of the risk of COVID-19 [24,25,26,27]. In an online survey of Chinese adolescents, results showed that perception of COVID-19 risk positively affected their understanding of and participation in social distancing behaviors [27]. Additionally, results of a survey of adults in Qatar found that the more highly they rated the danger of COVID-19, the more likely they were to socially distance [24]. Similarly, in a sample of adults in Hong Kong, a structural equation model revealed that perceptions of COVID-19 risk significantly affected compliance with protective measures (e.g., handwashing, social distancing) [25]. Further, researchers administered a survey to adults in Portugal and found that anxiety regarding COVID-19 and fear of death from COVID-19 significantly predicted protective behaviors, mediated by one’s perception of their own perceived risk [26].

Moreover, at the family level, the main determinant that has been examined in the context of protective behavioral changes since COVID-19 is low parental monitoring [21]. Among Swiss young adults, low parental monitoring was associated with lower COVID-19 protective behavior compliance [21]. However, family factors such as conflict and emotion expression were related with COVID-19 stressors [28]. Thus, it is of interest to determine how COVID-19 stressors affect such family factors, which thereby may influence protective behaviors against COVID-19 with the intention to reduce the spread of COVID-19 and associated stressors.

#### 1.1.3. The Current Study

The unprecedented, rapid spread of COVID-19 has negatively affected mankind. Middle school students are of particular interest when examining the impact of the COVID-19 pandemic because they are in a formative period for socioemotional development, and because they are not as mature as adults, making them more vulnerable to the effects of the current pandemic. Although middle school students are greatly affected by COVID-19, there are few studies looking at how COVID-19 affects them. In particular, we do not know how their behaviors and lives have changed since COVID-19. Thus, this study explores the relationships between possible indicators and protective behavior changes among middle school students since the COVID-19 pandemic began. This study seeks to (1) examine how individual factors such as age, gender and physical health influenced protective behavior changes since COVID-19; (2) investigate how family factors such as closeness and communication quality influenced protective behavior changes; and (3) explore how environmental factors such as levels of sanitation since COVID-19 and perceptions regarding the risk of COVID-19 influenced protective behavior changes since the beginning of the COVID-19 pandemic.

## 2. Methods

### 2.1. Participants and Sampling

Participants were recruited through an official online flatform used by public schools. The flatform is used as a communication tool between the schools and students and all students are able to receive notices from the school through the flatform. For this study, the target population was limited to middle school students enrolled in a public school in Gyeonggi province, the most populous area in South Korea. Participants were a convenience sample of middle school students. Data was collected from September to October 2020. Questionnaires were created in Google Forms and a link for the online survey was distributed through the public school’s online flatform. To protect students’ rights and improve the quality of the items, the questionnaires were evaluated and refined by experts, such as a middle school teacher. Participants received a $2 gift card as an incentive, and it took about twenty minutes to complete the survey. A total of three hundred fifteen four students participated. We excluded participants who declined consent to participate, either by themselves or by their caregivers. 26 respondents did not consent to participate so we excluded them in the final sample. As a result, the final sample included three-hundred twenty-eight participants. Even though this study does not contain any private identifiable information, this study was approved by the Institutional Review Board (#200810-1A). Further, given that the participants are not adults, a consent form was provided to both middle school students and their caregivers. Only middle school students whose caregivers also agreed with the participation engaged in this study. In addition, we did not collect any private information such as name, address, etc.

### 2.2. Measures

#### 2.2.1. Protective Behavior Changes Since COVID-19

This scale measured to what extent middle school students changed their behaviors since the emergence of the COVID-19 pandemic. Participants were asked if they had/were “decreased outdoor activities”, “reduced frequency of social gatherings”, “more careful about cleanliness”, and “increased time at home”. This measurement consisted of four items which were rated on a five-point Likert-type scale. Response options for all items ranged from 1 (strongly disagree) to 5 (strongly agree). All responses were summed with higher scores indicating greater behavioral changes since COVID-19. Cronbach’s α of behavioral changes since COVID-19 was 0.70 in this study (*M* = 16.85; *SD* = 2.82; ranging from 4 to 20).

#### 2.2.2. Individual Factors

Age and gender were included as determinants in this study. In addition, respondents reported on their health status, which was measured via the Health Status subscale [29,30]. This measure consists of five items which are rated on a five-point Likert-type scale. Middle school students responded to the following questions: “I am in good physical health”, “My body is in good physical shape”, “I am a well-exercised person”, “My body needs a lot of work in be in excellent physical shape”, and “My physical health is in need of attention.” The response options were as follows: Not at all characteristic of me; slightly characteristic of me; somewhat characteristic of me; moderately characteristic of me; and very characteristic of me. Two items were reverse-coded before analysis. We summed all items and higher scores indicated that respondents had greater physical health status. In this study, this scale had a Cronbach’s alpha of 0.67 (*M* = 16.23; *SD* = 3.63; ranging from 6 to 25).

#### 2.2.3. Family Factors

##### Family Satisfaction

The Family Satisfaction Scale was used to measure how satisfied respondents were with their family members [31]. This scale consists of ten items with a five-point Likert-type scale. For each item, participants were asked to indicate whether they were very dissatisfied, somewhat dissatisfied, generally satisfied, very satisfied, or extremely satisfied. Specific statements include the following: “The degree of closeness between family members”, “Your family’s ability to cope with stress”, “The quality of communication between family members”, and “Your family’s ability to resolve conflicts.” Each item was summed with higher scores indicating greater levels of family satisfaction. The Family Satisfaction Scale items in this study had a Cronbach’s alpha of 0.92 (*M* = 38.77; *SD* = 7.50; ranging from 12 to 50).

##### Subjective Poverty

Respondents reported levels of subjective poverty by answering “In your circumstances, do you consider your household’s economic status to be good or bad?” This question was derived from the Leyden Poverty Line [32]. The response options were as follows: very bad, bad, insufficient, sufficient, good, and very good. In this study, those who answered very bad, bad, or insufficient were regarded as being in poverty, while those who reported sufficient, good, or very good were considered as not being in poverty.

#### 2.2.4. Environmental Factors

##### Levels of Sanitation Since COVID-19

Middle school students indicated to what extent they had changed their daily sanitation activities since COVID-19. They were queried whether they “Wash hands frequently”, “Avoid touching own face”, “Do not share personal items”, or are “reluctant to go to crowded places due to hygiene problems.” The respondents indicated their levels of sanitation since COVID-19 by choosing one of the following: strongly disagree, disagree, neutral, agree, or strongly agree. Each item was summed, with higher scores indicating greater levels of sanitation since COVID-19. The Cronbach’s α of the five-point Likert-type scale was 0.71 (*M* = 16.42; *SD* = 2.72; ranging from 8 to 20).

##### Perceptions Regarding the Risk of COVID-19

Respondents stated to what extent they were aware of COVID-19. This scale consisted of four items which were rated on a five-point Likert-type scale with response options ranging from 1 (strongly disagree) to 5 (strongly agree). The specific items were as follows: COVID-19 is different than flu; [I] do not know when COVID-19 is gone, [COVID-19] damage[s] my health status, and [COVID-19] negatively influence[s] my daily life (e.g., by decreased frequency of dining out). The items were summed and higher scores indicated greater perception of risk regarding COVID-19. Cronbach’s α of this scale was 0.70 (*M* = 18.06; *SD* = 2.23; ranging from 10 to 20).

### 2.3. Analysis Strategy

**Figure 1:**
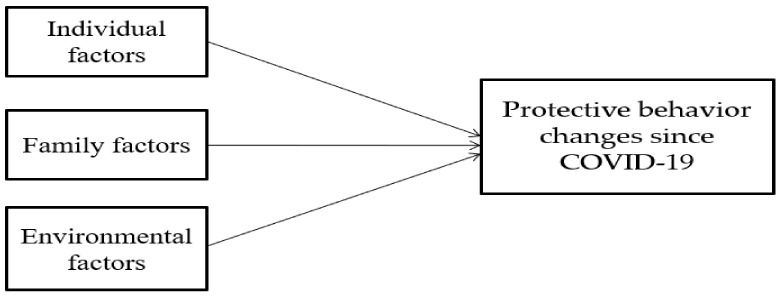
Research Design.

Analysis of variance and chi-squared tests were used to examine gender differences in individual, family, and environmental factors. A multiple linear regression was conducted to explore what factors influence protective behavior changes since COVID-19. Statistical Package for the Social Sciences (SPSS) 22.0 (IBM, Armonk, NY, USA) was employed to investigate the relationships between indicators and protective behavior changes since COVID-19. In the multiple linear regression model, individual factors were first considered, and then family factors and environmental factors sequentially addressed in the model. In other words, individual factors were included in model 1 while family factors were entered in model 2 with individual factors. Environmental factors were lastly added in model 3. Figure 1 shows the research design for the current study.

## 3. Results

Table 1 shows the descriptive statistics of variables used in the current study and gender differences in the dependent variable. There is a difference in protective behavior changes since COVID-19 between middle school boys and girls. Middle school girls had greater behavioral changes (17.20) than middle school boys (16.40) since the COVID-19 pandemic. Girls showed more decreased outdoor activities compared to boys (4.19 vs. 3.94), more reduced frequency of social gatherings (4.34 vs. 4.03), and more increased time at home (4.68 vs. 4.49). The average age of the middle school students was 14.4 years old. Slightly more than one-fourth of the total sample reported that they subjectively perceived being in poverty. The average scores for health status, family satisfaction, levels of sanitation since COVID-19, and perceptions regarding the risk of COVID-19 were 16.23, 38.77, 16.42, and 18.06, respectively.

Table 2 indicates which factors significantly influenced protective behavior changes since COVID-19 among middle school students. Model 1 includes individual factors and reveals that gender was associated with protective behavior changes since COVID-19 (*β* = 0.77, *p* < 0.05). When family factors were entered into model 2, girls were still more likely to have greater protective behavior changes since COVID-19 as compared to boys (*β* = 0.76, *p* < 0.05). Health status was negatively related to protective behavior changes since COVID-19 (*β* = −0.12, *p* < 0.01). Regarding family factors, family satisfaction was positively associated with protective behavior changes (*β* = 0.12, *p* < 0.001). Once COVID-19 environmental factors were included in model 3, gender, health status, and family satisfaction remained significant (β = 0.68, *p* < 0.05; *β* = −0.14, *p* < 0.001; *β* = 0.06, *p* < 0.01). For COVID-19 environmental factors, both levels of sanitation since COVID-19 and perceptions regarding the risk of COVID-19 were significantly related to protective behavior changes since COVID-19 (*β* = 0.27, *p* < 0.001; *β* = 0.35, *p* < 0.001).

## 4. Discussion

The principal purpose of the current study was to examine determinants of protective behavior changes since COVID-19 in the context of three dimensions: individual, family, and environmental. This study revealed how individual, family, and environmental determinants influenced protective behavior changes among middle school students in South Korea since the emergence of the COVID-19 pandemic. Even though all people, regardless of their demographics or socioeconomic status, have been greatly influenced by the unprecedented spread of COVID-19, middle school students are at particular risk because of the importance of the adolescent years for development [33]. Given that little is known about protective behavior changes since COVID-19 among middle school students, the present study contributes to understanding the determinants of COVID-19 protective behavior changes in this population. This study indicated that both individual and family factors were important to understand protective behavior changes. Further, levels of sanitation since COVID-19 and perception of risk regarding COVID-19 also influenced protective behavior changes among middle school students.

Individual factors that influenced protective behavior changes since COVID-19 among middle school students included gender and perceived health status. This study indicated that there is a gender difference in the protective behavior changes since the coronavirus outbreak. Middle school girls showed that they were more inactive since COVID-19 compared to middle school boys. More specifically, girls reported decreased outdoor activities, reduced frequency of social gatherings, and increased time at home. Generally, being inactive may be perceived in a negative way, but the meaning during the coronavirus pandemic could be understood to be more careful and cautious so as not to be infected by the virus. In general, adolescent boys are more likely than adolescent girls to engage in risky behaviors [34], which in the time of COVID-19, may manifest as engaging in social activities. Greater behavior changes during the coronavirus pandemic means that people are less likely to be infected, indicating that middle school boys should be educated to be more careful to avoid the spread of COVID-19. That greater health status was related to fewer protective behavior changes since COVID-19 demonstrated that healthy individuals may underestimate the risks of coronavirus and therefore continue to behave as they did before the pandemic. That is, healthy people may think that they are able to overcome the virus even if they are infected and think that the coronavirus is similar to the seasonal flu. However, given higher death rates around the world due to the virus and the unprecedented influence on the global economy and individuals’ lives [35], everyone should be very careful not to be infected, regardless of health status. Seemingly healthy people are not immune from coronavirus infections and negative consequences, but even if they have a mild infection, they can be a vector for transmitting the virus to the most vulnerable, such as older adults or those with underlying conditions that make them more at risk. Thus, even healthy middle school students must participate in preventive measures, such as social distancing, in order to protect themselves and other people.

This study confirmed that some family factors were also related to protective behavior changes since coronavirus emerged. Households’ economic resources were not related to such behavioral changes, while family satisfaction was associated with the behavioral changes. The specific protective behavior changes include reduced outdoor time with friends and increased time at home due to social distancing. If people have negative family situations, spending more time together at home due to COVID-19-related policies may lead to conflict and other negative outcomes [36]. On the other hand, middle school students who have good relationships with their family members may be more accepting of spending more time at home. That is, individuals with higher family satisfaction may be more likely to engage in such COVID-19 preventive practices by staying home with their family members, leading them to be more protected from coronavirus disease. However, middle school students who have more conflicts and disharmony with parents or siblings may be exposed to higher risks of COVID-19 through behaviors which may expose them to the virus. That is, improved relationships between children and parents or siblings can result in protective behavior changes related to social distancing, leading to decreased risks of coronavirus infection among middle school students.

It is also important to pay attention to environmental factors related to COVID-19 to understand protective behavior changes since the coronavirus disease emerged. This study revealed that higher levels of sanitation since COVID-19 and greater perception of risk related to COVID-19 results in greater protective behavior changes among middle school students. In other words, environmental changes due to the virus have an impact on middle school students’ behaviors. Middle school students who increase their sanitation behaviors may also be more likely to follow social distancing guidelines and therefore be at decreased risk for coronavirus infection. Thus, it is critical for middle school students to be educated about the importance of sanitary behaviors during the COVID-19 pandemic. This education on sanitary behaviors can be helpful to prevent the spread of coronavirus as middle school students change their behaviors to protect themselves from COVID-19. We recommend that this education includes the following: the importance of frequent hand washing; not touching one’s face; not sharing personal items; and avoiding going to crowded places. In addition, middle school students who better perceived the risks of COVID-19 showed greater changes in their daily behaviors, thereby lowering their potential exposure to coronavirus. As middle school students are still maturing and spend most of their time at home and school, the role of parents and teachers is important to increase their perceptions regarding the risk of COVID-19. That is, adults who are responsible for the health of middle school children should first understand the risks of COVID-19, because they are a primary source to teach the children about the risks of COVID-19 [23]. However, along with receiving information from parents and other adults, children are likely to access and retrieve information from social media such as Instagram, Facebook, Twitter, etc. [19]. Given that social media provides both true and false information [37], it is imperative to teach middle school children how to distinguish correct information about COVID-19 from inaccurate information.

## 5. Conclusions

This study indicated the importance of environmental factors to influence behaviors that decrease the risk of COVID-19 infection. After a year, the COVID-19 pandemic is still affecting people around the world; however, little is known about determinants of protective behavior changes among middle school students since COVID-19, which helps middle school students be at lower risk of being infected by COVID-19. This study contributes to literature related to COVID-19, particularly among middle school students, and suggests to consider three factors–individual, family, and environmental—in order to prevent middle school students from contracting and spreading the virus.

Even though this study sheds light middle school students’ protective behavior changes since the beginning of the COVID-19 pandemic, findings should be interpreted in the context of limitations. First, this study was conducted in South Korea, so behavioral changes among middle school students in other countries might be different from those in South Korea. Thus, cultural differences should be considered if the findings are to be applied to other settings. Second, additional factors can influence protective behavior changes since COVID-19, and we recommend that extra variables should be included in future studies. Third, this study focuses on middle school students regardless of disability status. Children with disabilities may have different determinants of protective behavior changes since the COVID-19 to consider. Therefore, we suggest that future studies focus on children with disabilities to identify if they differ from children without disabilities in terms of factors influencing protective behavior changes since the COVID-19 pandemic. Fourth, we calculated the sample size using a sample size calculator [38] with 5.24 confidence interval and 95% confidence level. The sample size needed in this study was 349; however, we recommend that an advanced power analysis or sample size estimation should be considered in future studies. Fifth, this survey needed to be completed during September and October 2020. Due to limited time and budget, we chose a convenience sample.

## Figures and Tables

**Table 1:** Descriptive Statistics for Variables Included in the Study.

Variable	Boys (*n* = 146)	Girls (*n* = 182)	Total	*p*
	% or Mean (SD)	% or Mean (SD)	(*n* = 328)	
Protective behavior changes since COVID-19	16.40 (3.25)	17.20 (2.38)	16.85 (2.82)	*
Individual factors				
Age	14.41 (.74)	14.37 (1.19)	14.39 (1.01)	
Health status	16.52 (3.76)	15.99 (3.53)	16.23 (3.63)	
Family factors				
Subjective poverty	28.1%	27.5%	27.7%	
Family satisfaction	38.95 (7.14)	38.63 (7.80)	38.77 (7.50)	
Environmental factors				
Levels of sanitation since COVID-19	16.51 (2.94)	16.35 (2.52)	16.42 (2.72)	
Perceptions regarding the risk of COVID-19	17.90 (2.30)	18.19 (2.18)	18.06 (2.23)	

Note: * *p* < 0.05; Significant difference between males and females.

**Table 2:** Regression Results of Unstandardized Coefficients (standard error), Predicting Protective Behavior Changes Since COVID-19.

Variables	Protective Behavior Changes Since COVID-19					
	Model 1		Model 2		Model 3	
(Constant)	17.05 (2.38)		15.03 (2.30)		4.66 (2.46)	
Individual factors						
Gender (girl)	0.77 (0.31)	*	0.76 (0.30)	*	0.68 (0.27)	*
Age	0.01 (0.15)		0.07 (0.15)		0.06 (0.14)	
Health status	−0.05 (.04)		−0.12 (0.04)	**	−0.14 (0.04)	***
Family factors						
Subjective poverty			−0.30 (0.34)		−0.30 (0.31)	
Family satisfaction			0.12 (0.02)	***	0.06 (0.02)	**
Environmental factors						
Levels of sanitation since COVID-19					0.27 (0.06)	***
Perceptions regarding the risk of COVID-19					0.35 (0.06)	***

Note. * *p* < 0.05. ** *p* < 0.01. *** *p* < 0.001.

## Data Availability

The data presented in this study are available on request from the corresponding author. The data are not publicly available due to ethical reasons.
